# Infrared Sensor-Based Temperature Control for Domestic Induction Cooktops

**DOI:** 10.3390/s140305278

**Published:** 2014-03-14

**Authors:** Javier Lasobras, Rafael Alonso, Claudio Carretero, Enrique Carretero, Eduardo Imaz

**Affiliations:** 1 Applied Physics Department, University of Zaragoza, C/Pedro Cerbuna, 12, 50009 Zaragoza, Spain; E-Mails: ralonso@unizar.es (R.A.); ccar@unizar.es (C.C.); ecarre@unizar.es (E.C.); 2 Global Cooking Product Division, Induction Cooktop Development, Bosch and Siemens Home Appliances Group, Avenida de la Industria, 49, 50016 Zaragoza, Spain; E-Mail: Eduardo.Imaz@bshg.com

**Keywords:** non-contact temperature measurement, infrared sensor, radiation detector, induction heating, temperature control

## Abstract

In this paper, a precise real-time temperature control system based on infrared (IR) thermometry for domestic induction cooking is presented. The temperature in the vessel constitutes the control variable of the closed-loop power control system implemented in a commercial induction cooker. A proportional-integral controller is applied to establish the output power level in order to reach the target temperature. An optical system and a signal conditioning circuit have been implemented. For the signal processing a microprocessor with 12-bit ADC and a sampling rate of 1 Ksps has been used. The analysis of the contributions to the infrared radiation permits the definition of a procedure to estimate the temperature of the vessel with a maximum temperature error of 5 °C in the range between 60 and 250 °C for a known cookware emissivity. A simple and necessary calibration procedure with a black-body sample is presented.

## 1. Introduction

Induction cooktops are becoming one of the most popular appliances due to their safety, cleanliness, and high cooking performance [1–3]. In these system, the vessels are heated up by two dissipation phenomena: the induced currents and the ferromagnetic losses, both of them originated by varying magnetic fields generated by alternating medium frequency currents (20 to 100 kHz) flowing through a planar coil placed below the glass-ceramic surface [4,5] where the vessel is placed. The current frequency supplied by the power electronics depends on the power level selected by the user.

In commercial induction hobs, the control system is based on a closed-loop control [6–10], which adapts the power supplied to the cookware depending on the selected level by the user and safety conditions. However, the temperature in the cookware is a hidden variable to the control because no temperature probe can be placed in contact with the vessel due to product requirements. The temperature estimation system has to be located below the cooking surface as well as the inductor coils and the power electronics, thus, the temperature measurements are influenced by the effect of the cooking surface which has to be compensated in order to obtain an accurate temperature value of the vessel. It should be noted the importance of this parameter as a control variable in order to achieve advances features for domestic induction appliances, for example, assisted or automatic cooking.

High performance measurement systems would consist on temperature sensors placed inside the cookware, but this solution is not user-friendly. Alternative solutions have been proposed in order to overcome this drawback. One of those consists of a temperature measurement system based on a thermistor located under the glass-ceramic where the steady-state cookware temperature is assumed equal to the temperature of the in-contact glass [11,12]. The proposed system constitutes a simple and cost-effective solution, but it possesses some disadvantages, mainly due to the thermal inertia of the glass-ceramic, which introduces attenuation and time delays between the temperature of the cookware and the temperature of the glass. This effect becomes very critical in rapid heating transients due to the large difference between the measured temperature in the glass and the temperature in the cookware which implies that the cookware can reach high temperatures before the temperature sensor detects this situation. Additionally, this proposed system exhibits a strong dependence of the cookware-glass heat transfer due to the variability in the air gap between the cookware and the glass which influences in the dynamic of the measurement procedure. Other system based on inductive sensing [13] or radiation thermometry have been proposed in order to avoid these problems. This paper is focused on the latter one. Optical infrared (IR) sensors of different technologies, for example, photo-resistors [14], thermopiles [15–18], in array format for infrared computer vision [19–21] and including optical fibers [22] have been analyzed by many authors for other applications. However, the systems based on the infrared radiation detection by photodiodes are expected to achieve the best performance because of their rapid response, reduced cost, as well as the industrial applications of this technique in the cooking appliances industry.

The aim of this work is to describe the main features of a temperature control system based on an IR temperature sensor proposed in preceding works [23,24] as well as to provide the experimental verification to validate the performance of this cost-effective solution.

**Figure 1.**
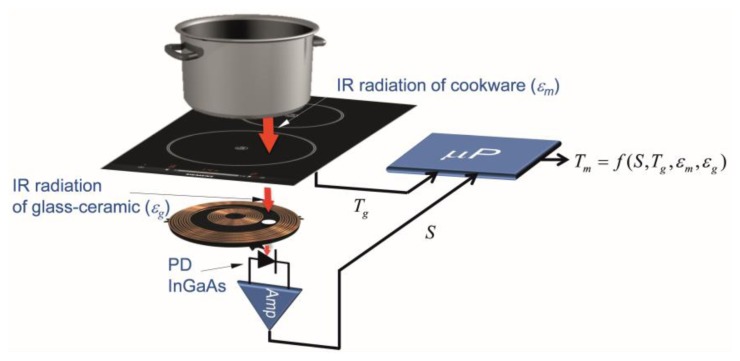
Cookware temperature measurement system based on an IR thermometry system which includes IR photodiode (PD), electronic amplifier and algorithm to estimate the temperature.

The temperature measurement system, shown in Figure 1, is based on the detection of the infrared light radiated by the bottom of the cookware and spectrally weighted by the glass-ceramic optical transmittance. The signal detected by the infrared photodiode (PD) depends on the temperature of the cookware, *T_m_*, the temperature glass-ceramic, *T_g_*, placed below the cookware, and the emissivities of the cookware, *ε_m_*, and the glass-ceramic, *ε_g_*, respectively. The power control system employs the measured temperature in order to adapt the power supplied by the inductor coil. The rapid response of the proposed system to temperature changes in the cookware is suitable to use to control the temperature of the cookware in rapid heating systems, for instance, induction cookers.

The paper is organized as follows: Section 2 introduces some theoretical background about the operational characteristics of the IR sensor depending on the temperatures and emissivities of the cookware and the glass-ceramic, respectively. Section 3 describes the electronic implementation of the proposed measurement system as well as the closed-loop power control and the calibration procedure of the IR sensor applied to compensate the effect of the glass-ceramic. Section 4 provides several experimental results in order to evaluate the main features of the proposed system. Finally, in Section 5 the main conclusions are drawn.

## 2. Analysis of IR Temperature Sensor

The temperature sensor is based on the detection of the infrared radiation by an extended range InGaAs PIN photodiode with a spectral responsivity up to 2,600 nm. A detailed analysis of the theoretical background given in the following subsections can be found in [24].

### 2.1. Infrared Signals in Domestic Induction Hobs

**Figure 2.**
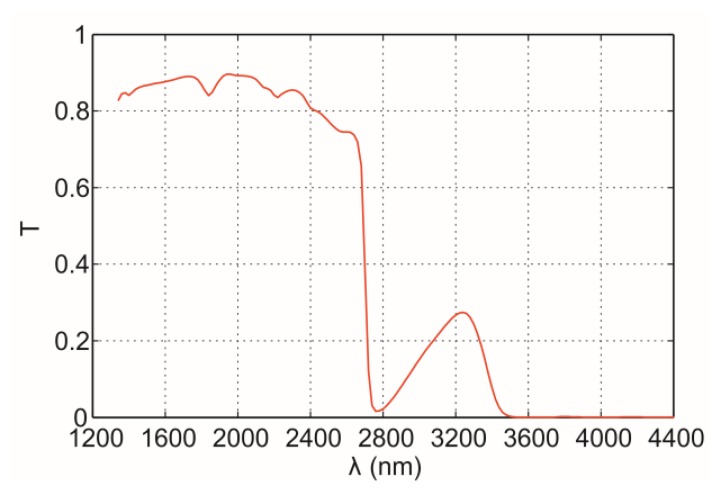
The four components of the thermal radiation of pan bottom/glass-ceramic system.

The model to analyze the thermal radiation of a metallic surface, (the bottom of the cookware), considered as a grey body of spectral emissivity, *ε_m_*, at the temperature, *T_m_*, in contact with a glass-ceramic top at the temperature, *T_g_*, is shown in Figure 2. The glass-ceramic radiation contribution has to be taken into account because it also emits additional infrared radiation to that emitted by the cookware and transmitted by the glass-ceramic [25,26].

The total emission of the cookware/glass-ceramic system can be modeled through the addition of four signal contributions, as shown in Figure 2. The first contribution belongs to the radiation which arrives from the bottom of the cookware through the glass slab. The second one is the radiation emitted by the glass. The third contribution is the emerging radiation that results from adding the multiple reflections and transmitted beams emitted by the bottom of the cookware. The fourth contribution is the addition of the reflected and the transmitted radiation emitted by the glass-ceramic toward the bottom of the cookware. The total spectral emissive power emitted by the cookware-glass system in the normal direction, is:

(1) $$E_{⊥}(\lambda ,T_{g},T_{m})=E_{⊥,1}(\lambda ,T_{m})+E_{⊥,2}(\lambda ,T_{g})+E_{⊥,3}(\lambda ,T_{m})+E_{⊥,4}(\lambda ,T_{g})$$

The four contributions to *E*_⊥_ (*λ*,*T_g_*,*T_m_*) can be expressed as:

(2) $$\begin{array}{ll} E_{⊥,1}(\lambda ,T_{m}) & =T{ɛ}_{m}\frac{E_{BB}(\lambda ,T_{m})}{\pi }d\Omega \\ E_{⊥,2}(\lambda ,T_{g}) & ={ɛ}_{g}(\lambda )\frac{E_{BB}(\lambda ,T_{g})}{\pi }d\Omega \\ E_{⊥,3}(\lambda ,T_{m}) & =T{ɛ}_{m}\frac{E_{BB}(\lambda ,T_{m})}{\pi }\frac{R(1-{ɛ}_{m})}{1-R(1-{ɛ}_{m})}d\Omega \\ E_{⊥,4}(\lambda ,T_{g}) & ={ɛ}_{g}(\lambda )\frac{E_{BB}(\lambda ,T_{g})}{\pi }\frac{T(1-{ɛ}_{m})}{1-R(1-{ɛ}_{m})}d\Omega \end{array}$$

where *T* and *R* are the optical transmittance and reflectance of the glass-ceramic material, respectively, which have been assumed constants in the spectral region of interest, and *E_bb_ (λ*,*T)* is the hemispherical spectral emissive power of a perfect black body following the Planck's law [27–29]:

(3) $$E_{BB}(\lambda ,T)=\frac{C_{1}}{\lambda^{5}\left(e^{C_{2}/\lambda T}-1\right)}$$

**Figure 3.**
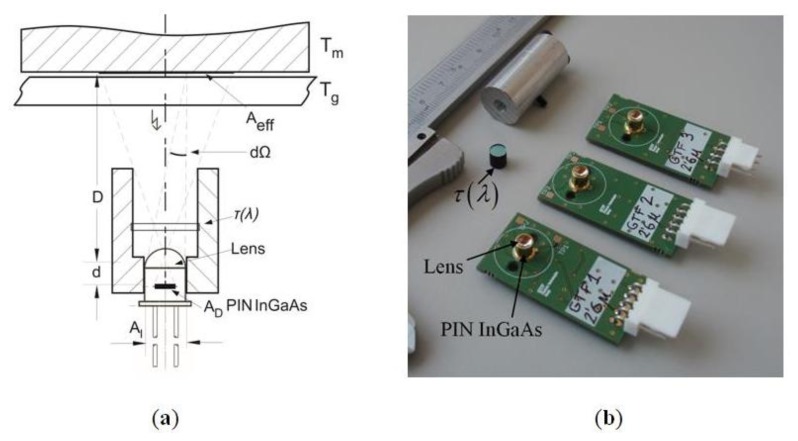
Total transmittance, *T*, of dark ceramic-glass. The spectral bandwidth of interest in the measurement procedure ranges from 1,200 to 2,600 nm.

Figure 3 shows the typical transmittance spectrum, *T*, of conventional dark glass-ceramic top. Infrared radiation from metallic surface with wavelength above 2,600 nm is attenuated by the glass-ceramic material. Note that the use of extended photodiodes is adequate to detect IR radiation emitted by this kind of material.

### 2.2. Long-Range Single-Band Thermometry IR System

**Figure 4.**
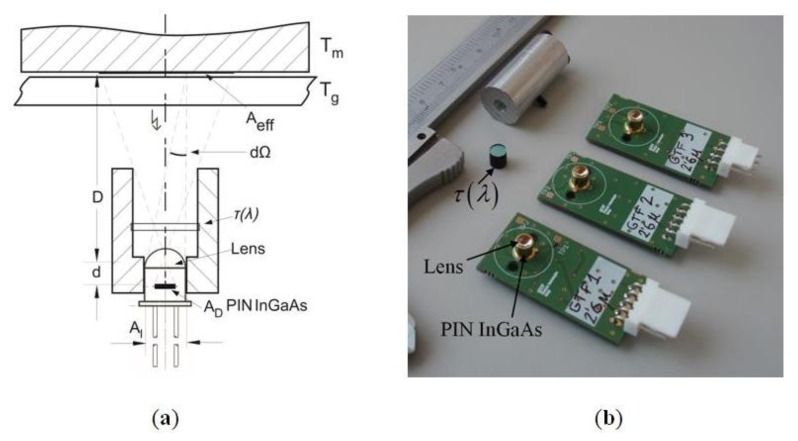
(**a**) Optical system, including dimensions, geometry, effective area of the radiation and the solid angle subtended by the lens. D = 30 − 33 mm, *d* = 3.6 mm, *A_l_* = *20* mm^2^ and *A_D_* = 0.78 mm^2^ (1 mm diameter); (**b**) Implemented optical system, including PIN InGaAs, filter *τ*(*λ*) and lens.

The optical temperature sensor system is shown in Figure 4a. The radiation is detected in a spectral band by a long-range IR InGaAs PIN photodiode with responsivity ℜ(*λ*), detection area *A_D_*, and placed at the focal point of the lens. Visible radiation from the environment is blocked by a high-pass optical filter with a cut-off wavelength of 1,200 nm and flat transmittance *τ*_0_ in the band from 1,200 to 2,600 nm. Figure 4b shows implemented optical system.

Assuming the independency with respect to the wavelength of the emissivity, the reflectance and the transmittance of the glass-ceramic and the cookware (grey body approach) in the region from 1,200 to 2,600 nm, the output voltage, *S*, due to the photo-current generated by a PIN can be expressed as [24]:

(4) $$S=\tau_{0}G\frac{d\Omega }{\pi }A_{\text{eff}}\left[T{ɛ}_{m}\left(1+\frac{R(1-{ɛ}_{m})}{1-R(1-{ɛ}_{m})}\right)f(T_{m})+{ɛ}_{g}\left(1+\frac{T(1-{ɛ}_{m})}{1-R(1-{ɛ}_{m})}\right)f(T_{g})\right]$$

where:

(5) $$f(T)=\int_{1200}^{2600}E_{BB}(\lambda ,T)ℜ(\lambda )d\lambda$$

and:

(6) $$\begin{array}{ll} A_{\text{eff}}=\frac{D}{d}A_{D}; & d\Omega =\frac{A}{{\left(D+d\right)}^{2}} \end{array}$$

The expression *f(T)* can be regarded as *f(T)* = *aT^q^*, then:

(7) $$S=\tau_{0}G\frac{A_{1}A_{D}D}{\pi d{\left(D+d\right)}^{2}}\left[T{ɛ}_{m}\left(1+\frac{R(1-{ɛ}_{m})}{1-R(1-{ɛ}_{m})}\right)aT_{m}^{q}+{ɛ}_{g}\left(1+\frac{R(1-{ɛ}_{m})}{1-R(1-{ɛ}_{m})}\right)aT_{g}^{q}\right]$$

The temperature *T_m_* can be derived from the preceding expression with dependence on the output signal, *S*, the emissivity of the cookware, *ε_m_*, the temperature, *T_g_*, the transmittance, *T*, and the reflectance, *R*, of the glass-ceramic, as it is given as follows:

(8) $$T_{m}={\left[\frac{\frac{S}{\tau_{0}aG}\frac{\pi d{\left(D+d\right)}^{2}}{A_{1}A_{D}D}-{ɛ}_{g}\left(1+\frac{R(1-{ɛ}_{m})}{1-R(1-{ɛ}_{m})}\right)T_{g}^{q}}{T{ɛ}_{m}\left(1+\frac{R(1-{ɛ}_{m})}{1-R(1-{ɛ}_{m})}\right)}\right]}^{1/q}$$

where the emissivity of glass-ceramic, *ε_g_*, can be derived from the total reflectance, *R*, and transmittance, *T*, of the glass slab, according to the well known expression:

(9) $${ɛ}_{g}=1-R-T$$

## 3. Electronic Implementation and Temperature Control System

The implementation of the IR sensor electronics and temperature control system are explained as follows:

### 3.1. Electronics of IR Sensor

Previous works demonstrate the usefulness of IR thermometry for cooking purposes within the range of frying temperatures ranging from 140 to 180 °C [24]. Unfortunately, not so good temperature estimation was achieved at temperatures below 110 °C because the photocurrent of a standard InGaAs PIN PD is comparable to the noise of the associated electronics. However, the proposed IR system is designed to measure temperatures ranging from 60 to 250 °C, therefore, an infrared detector with a long wavelength cut-off at 2,600 nm is needed.

InGaAs PIN photodiodes are photovoltaic detectors having p-n junctions just like Si photodiodes. InGaAs PIN photodiodes possesses a wider sensitivity wavelength range than Si photodiodes due to their smaller energy gap. Consequently, infrared detectors with spectral response ranges with a long wavelength cut-off at 2,600 nm are available in the market. In this work, the InGaAs PIN J23T E2-66C-R 01M-2.6, supplied by Teledyne Judson Technologies (Montgomeryville, PA, USA) was selected, with enhanced spectral responsivity up to 2,600 nm.

The output signal provided by the detector is low (a few nanoamperes), therefore, the amplification and filtering of the signal is needed prior to the processing of the signal. With this purpose, a wide variety of amplifier configurations can be selected based on noise, bandwidth, offset, and linearity.

**Figure 5.**
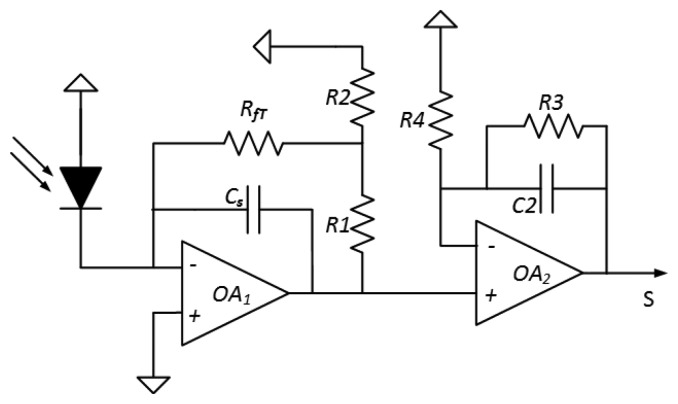
Dual stage transimpedance amplifier with resistive-tee feedback.

The system represented in Figure 5 includes an InGaAs PIN photodiode and a dual-stage circuit. The first stage is a tee-transimpedance amplifier topology [30–33], which transforms the photocurrent (current generated by the PD) into voltage. The most popular design approach to achieve high precision current-to-voltage conversion is the circuit composed of an operational amplifier network with a resistor in the feedback loop. In particular, the resistor tee-network still uses the fundamental concept of a resistive feedback loop to perform the current-to voltage conversion. In this circuit, *R_1_* and *R_2_* form a voltage divider which represents a fraction of the output voltage to *R_fT_*. Generally, *R_1_* and *R_2_* are small compared to *R_fT_*, thus, the effective feedback resistance *R_eq_* is given by:

(10) $$R_{\text{feq}}=\left(R_{fT}+R_{1}+\frac{R_{1}R_{fT}}{R_{2}}\right)$$

Note that highly effective feedback loop can be built relatively small values of resistances [34] which is useful in high-gain systems that would otherwise be limited by the effect of stray capacitance on *R_fT_*.

An additional benefit of the resistive tee-network is the reduction by a factor of (1 + *R_1_*/*R_2_*) in the output offset arising from the bias current requirement of the amplifier, but, at the expense of a proportional increase in the output error from the amplifier offset voltage [35]. The operational amplifier selected to implement the electronics are the low offset operational amplifier AD8639 from Analog Devices because provides high performance results.

The second stage post-amplifies and filters the output signals of the first stage with a very low cut-off frequency filter because the signal of interest is *dc* with slow variations over time due to the temperature of the cookware changes slowly. Usually, the transimpedance amplifier is connected to a low pass filter to further reduce the wideband noise in the rejected bandwidth. A single pole, low-pass filter improves the dynamic range of the transimpedance amplifier by a factor of 4 or 5 dB.

Finally, the output voltage, *S*, of the electronics is given by the following expression:

(11) $$S=I_{ph}\left(R_{ft}+R_{1}+\frac{R_{1}R_{ft}}{R_{2}}\right)\left(1+\frac{R_{3}}{R_{4}}\right)$$

where *I_ph_* represents the photocurrent generated by the PD. The first gain factor represents the current-to-voltage conversion in the transimpedance stage with *R_fT_* = 510 kΩ, *R_1_* = 1 kΩ, *R_2_* = 100 Ω, *R_3_* = 100 kΩ, *R_4_* = 100 kΩ; thus, the conversion factor of the transimpedance stage is G_1_ = 5.5 × 10^6^ V/A and the gain factor of the second one is *G*_2_ ≈ 2.

The cut-off frequency of the first stage is limited by the gain bandwidth product due to the high gain factor. The capacitor *C_s_* is added for stability purposes. The cut-off frequency of the second stage has to be low enough in order to filter any kind of noise, but it should be as low as it is necessary to follow the evolution of the temperature over time. The cutoff frequency is around 16 Hz for *C*_2_ = 100 nF and *R*_3_ = 100 kΩ.

### 3.2. Experimental IR Temperature Control System for Induction Appliances

**Figure 6.**
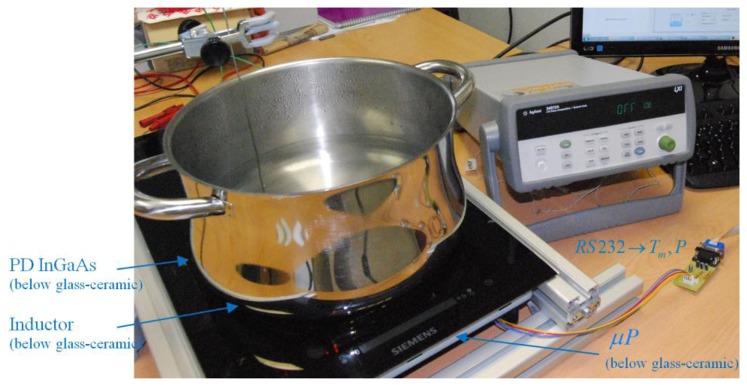
Experimental setup.

The experimental setup shown in Figure 6 has been built to test the performance of the preceding IR sensor under actual operational conditions of a domestic induction cooker.

#### 3.2.1. Hardware Implementation

**Figure 7.**
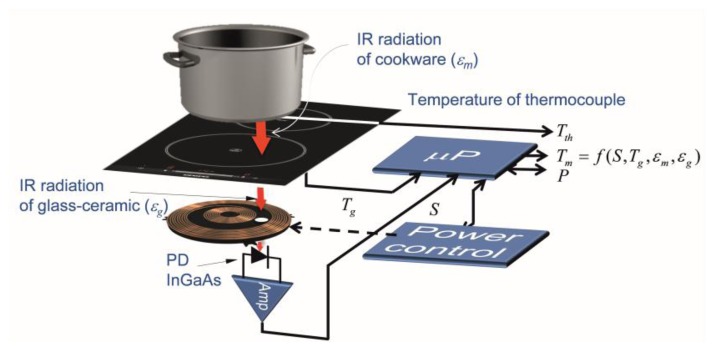
Diagram of the temperature control system.

Figure 7 shows the block diagram of the temperature control system. The IR sensor is placed below the glass-ceramic slab. The temperature of the vessel is monitored by a thermocouple. The pan is heated up by the induction heating system which generates ringlike temperature distributions [6,8,36], where the hot spots are located in the middle positions of the ring.

In steady state at low power levels, the temperature distribution is more uniform than in heating transients. However, the temperature distribution is non-uniform at high power levels. To avoid this phenomenon in these kinds of heaters, the IR sensor and the thermocouple must be located at the same radial point. Radial point with maximum temperature has been selected.

The measurement procedure has been automated in order to acquire a large amount of experimental data. A data acquisition system (Agilent 34972A, Agilent Technologies Inc, SANTA CLARA, CA, USA) is used to store the information given by the thermocouple. Information relating to the glass-ceramic temperature and IR output voltage is obtained from the microprocessor. Microprocessor PIC24EP64GP206 of Microchip Technology (Chandler, AZ, USA) has been used, with 12-bit ADC and a sampling rate of 1 Ksps. A proportional-integral control is applied to control the output power level in order to reach the target temperature.

#### 3.2.2. Software

**Figure 8.**
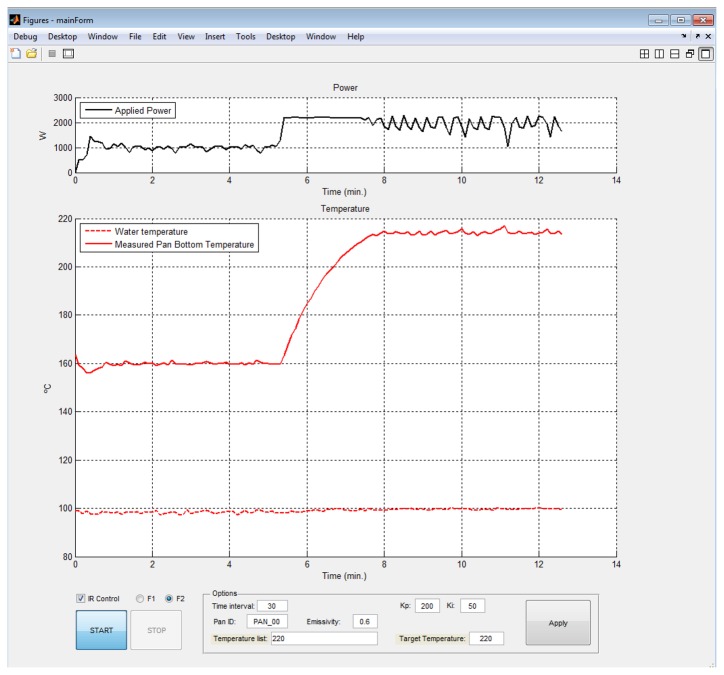
Software interface module.

The main purpose of the software is to control the data acquisition process and to manage the acquired data. MATLAB is adopted as the programming language. The software of the monitoring system permits the configuration of the system and displays different data. Figure 8 shows the MATLAB user friendly interface module developed for displaying the electrical power, the temperature measurements and the estimation of the IR sensor temperature. The interface is suitable to select the parameters of the proportional-integral-controller and the target temperature. Afterwards, the data acquisition system runs automatically.

#### 3.2.3. Calibration Procedure

The calibration of the IR sensor is required to obtain accurate temperature measurements in actual induction heating systems. Parameters *a* and *q* given in Equation (7) have been estimated from experimental measurements using a sample with constant emissivity in the wavelength band ranging between 1,200 and 2,600 nm.

**Figure 9.**
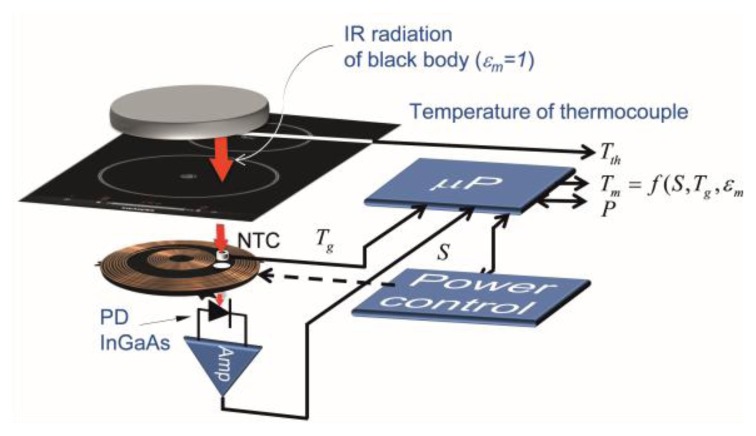
Experimental setup to measure the black-body temperature, *T_th_*, with a thermocouple.

**Figure 10.**
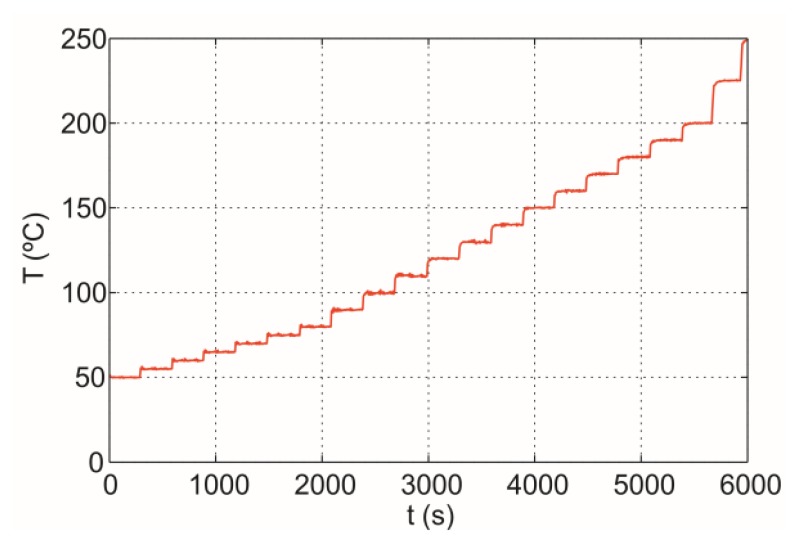
Black-body temperature, *T_th_*, measured with a thermocouple applying a given thermal cycle.

A black-body sample is heated by the induction heating system, and the temperature of the bottom of the sample, *T_m_*, and the glass-ceramic, *T_g_*, are registered simultaneously, as we can see in Figure 9. The temperature of the sample is varied from room temperature to 250 °C. Figure 10 shows the temperature of the bottom of the black-body measured with a thermocouple, *T_th_* Temperature of the black-body bottom is stabilized over intervals of 250 s.

**Figure 11.**
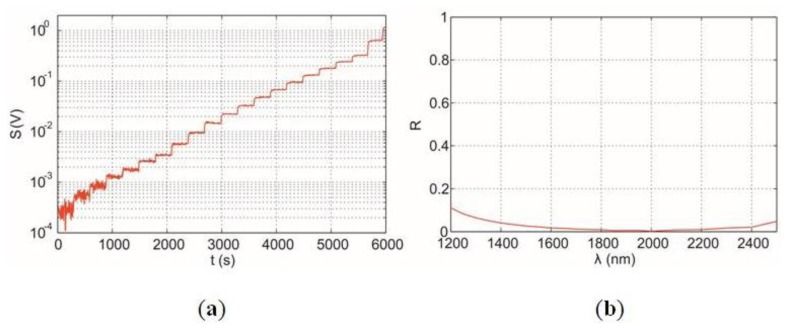
(**a**) Measured output voltage *S* from the black-body infrared radiation; (**b**) Spectral reflectance of ferromagnetic black body used in calibration of sensor. Spectral emissivity (*ε* = 1 − R) can be considered almost equal to the unit in the range of interest.

Figure 11a shows the measured output voltage, *S*, of sensor measured simultaneously with previous measurement. The black-body sample is built with a special multilayer structure deposited on a ferromagnetic steel disc using Physical Vapour Deposition (PVD) technique. The total reflectance is measured with a Vis-IR spectrophotometer equipped with an integrating sphere, shown in Figure 11b.

**Figure 12.**
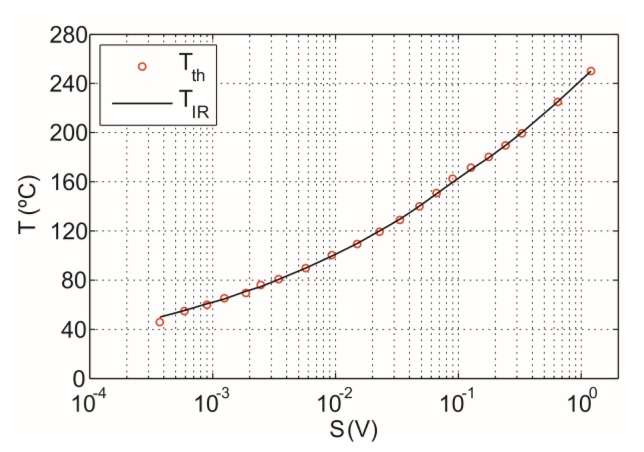
Calibration curve obtained from theoretical expressions.

The temperature in the cookware, Tm, is calculated applying Equation (8). Figure 12 shows the calibration curve obtained from experimental measurement. A good fitting for the black-body is achieved.

After the calibration of the system was performed according to the previously described procedure, new measurements of the black-body in the temperature ranging from 50 to 250 °C are carried out.

**Figure 13.**
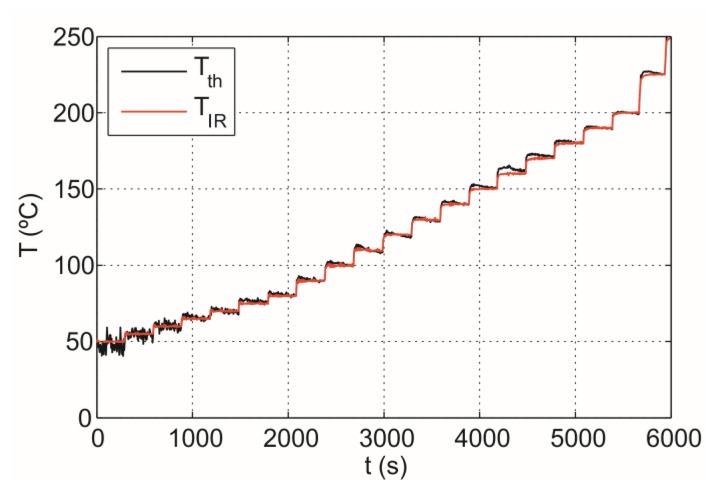
IR sensor precision for black-body sample measurment in the temperature range from 50 to 250 °C.

Figure 13 shows the comparison between the IR sensor measured temperature, *T_IR_*, and the temperature measured by the thermocouple, *T_th_*.

## 4. Results and Discussion

Several test and measurements are carried out in order to validate the reliability and accuracy of the experimental measurements as well as the applicability of IR sensor.

### 4.1. Response

First of all, the IR sensor applied to the domestic induction heating cooktop is tested at temperatures ranging from 60 to 250 °C was carried. In that case, a metal disk sample with emissivity *ε_m_* = 0.6 acts as the induction load. The temperature of the metal disk sample, *T_m_*, is measured by a thermocouple attached to its base.

**Figure 14.**
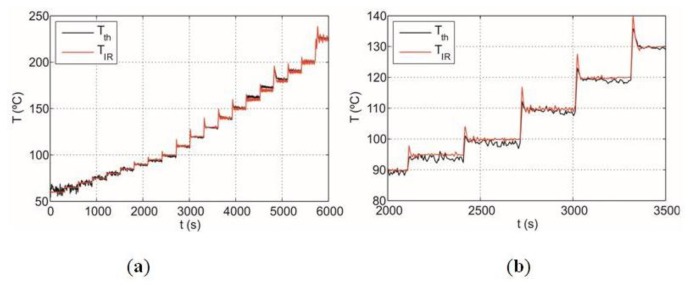
Temperature measured with a thermocouple and the infrared sensor, respectively, for a metal disk sample: (**a**) temperature ranging from 60 to 250 °C; (**b**) temperature ranging from 90 to 130 °C.

Figure 14a shows temperature measured with the thermocouple, *T_th_*, and the infrared sensor temperature, *T_IR_*, stabilized by the control. The maximum temperature error of 5 °C is obtained in the range between 60 and 250 °C whereas the average error of the measurement is around 1.5 °C. It should be noted that the proposed IR system provides accurate temperature measurement and power controlling below 60 °C.

**Figure 15.**
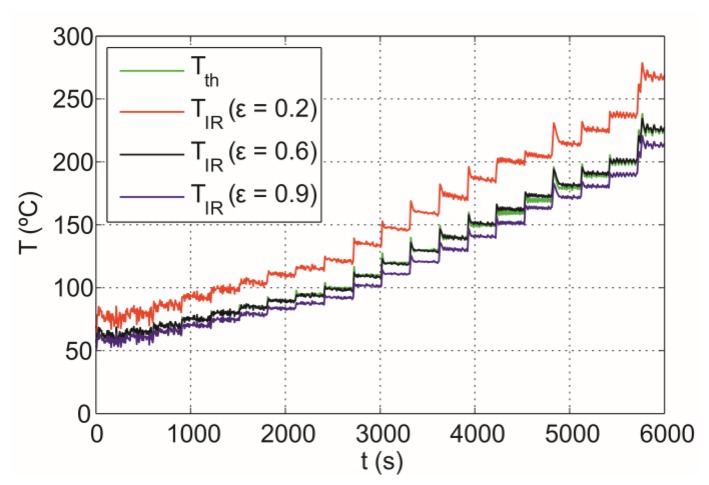
Estimated IR sensor temperature for different sample emissitivy values.

In a real system, the emissivity of the cookware is not well-known. Cookwares have typical emissivities in the range from 0.2 to 0.9. Estimated errors for the IR sensor temperature assuming different sample emissivities are shown in Figure 15. The temperature is overestimated at low-emissivities whereas the system overestimates the temperature at high-emissivities. In conclusion, the maximum absolute error is around 20 °C when the emissivity of the cookware is not known.

As a consequence, accurate estimation of the emissivity of the cookware is required to achieve accurate temperature measurement for all types of cookware because, in that case, the temperature can be determined with a precision of within 5 °C, as shown in Figure 14b.

Determination or compensation of emissivity, in real time, can be performed in different ways. First, by multi-wavelength infrared thermometry technique [37–40], second, by a direct measurement of the total reflectance, and, finally, through the use of compensation techniques based on radiation exchange between surfaces [41–43].

### 4.2. Water Pre-Boiling and Boiling Point Temperature Control in an Induction Heating Hob

Although the efficiency of the power electronics is very high the waste of energy in the cooking process highly decreases the energy efficiency of induction cookers. Improvements in the energy efficiency during the cooking process could be therefore achieved by means of accurate pot temperature control. Control system [44] for the temperature of food during the cooking and automatic detection system of the boiling point [45,46] are also advantageous. For instance, pot temperature control ensures correct food cooking minimizing the cooking time as well as avoids the hazard to reach excessive temperatures, which can imply the burning of the food.

**Figure 16.**
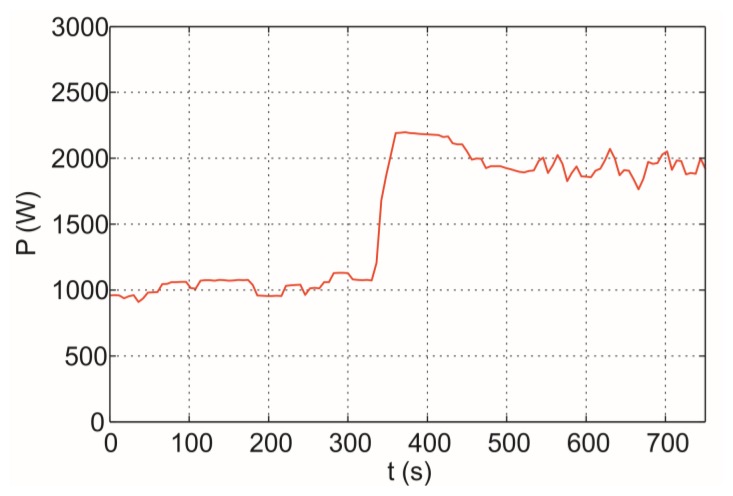
Electrical power at pre-boiling and boiling points.

The relationship between the IR sensor temperature and water temperature depends on the boiling conditions determined, among others, by the type of container, volume of water, applied heater power. Figure 16 shows electrical power at same pre-boiling (97.5 °C) and boiling (100 °C) points under the operation conditions shown in Figure 8. At boiling point, the electrical power clearly increases, thus, the detection of the boiling point implies energy savings.

Both IR sensor and electrical power information could be very useful to define an automatic boiling point detection algorithm. The thermal transmission properties of the cookware at the boiling process can be extracted from the bottom cookware temperature and the maximum electrical power delivered by the cooktop. The thermal power flow can be derived from electrical the power consumption due to the high efficiency [47] of induction hobs.

## 5. Conclusions

In this work, a precise temperature control system based on IR thermometry has been presented. The theoretical analysis of the system includes an algorithm to discount glass-ceramic contribution from the total signal which allows us to obtain the temperature of the cookware with a maximum temperature error of 5 °C in the range between 60 and 250 °C for a known cookware emissivity.

A real-time feedback control of the temperature has been implemented by means of induction hob electrical power. A simple and necessary calibration procedure with a black-body sample is presented.

The accuracy of our model has been tested and confirmed with measurements performed with the proposed system. It has been proved that the IR sensor works properly to stabilize the temperature in the range from 60 to 250 °C in the cookware heats up by commercial domestic induction cookers. Table 1 shows main characteristics of the IR temperature control system.

The output signal levels of the proposed system exhibit a moderate dependence on the emissivity of the cookware. Then, it is necessary to know accurately the emissivity of the cookware in order to achieve accurate temperature measurements for all types of cookware. Future works will be oriented in this way, because, by estimating the emissivity of cookware, the temperature can be determined with a precision of within 5 °C.

## Figures and Tables

**Table 1.** Main system characteristics.

Parameter	Value	Units
Wavelength	1,200–2,600	nm
Power control	0–2,200	W
Temperature range	60–250	°C
Precision *	±5	°C
Time response	_10_^−3^	s

Microprocessor Microchip PIC24EP64GP206		

Sampling rate	1	Ksps
ADC	12	bits

* Precision for a known cookware emissivity.
